# Extraction optimization by using response surface methodology and purification of yellow pigment from *Gardenia jasminoides* var. *radicans* Makikno

**DOI:** 10.1002/fsn3.2046

**Published:** 2020-12-04

**Authors:** Jun Wu, Jiangtao Zhang, Xin Yu, Yue Shu, Siyu Zhang, Yinglao Zhang

**Affiliations:** ^1^ School of Life Science Anhui Agricultural University Hefei China

**Keywords:** antioxidant activity, *Gardenia jasminoides* var. *radicans* Makikno, Macroporous resins, response surface methodology, yellow pigment

## Abstract

*Gardenia jasminoides* var. *radicans* Makikno* *contains rich gardenia yellow pigment (GYP). In this study, the process of pigment extraction was optimized based on a Box–Behnken design (BBD) and response surface methodology (RSM). The absorbance and antioxidant activity (AA) were considered as responses. The result showed that the optimal extraction conditions were ethanol concentration 65.10%, liquid/solid ratio 10:1 ml/g, extraction time 59.85 min, and extraction temperature 60.04℃ for the maximal response values of absorbance (0.79) and AA (91.30%), respectively. Crude GYP was purified by the 13 different resins. The result showed that BJ‐7514 was suitable for purifying GYP with the absorption ratio of 95.4%. Moreover, the 80% of ethanol eluent is applicable on the BJ‐7514 with the desorption ratio of 91.93%. The major component of GYP (Crocin‐3) was isolated and identified from the purified GYP.

## Graphical abstract

Heat‐aided solvent extraction of *Gardenia jasminoides* var. *radicans* Makikno yellow pigment was reported. Extraction conditions were optimized by response surface methodology. The resin BJ‐7514 was suitable for purifying the yellow pigments from *Gardenia jasminoides* var. *radicans* Makikno.

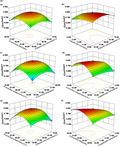

## 1 INTRODUCTION

Color is a critical factor in consumers choosing favorite food. During food processing and storage, the original appearance of food is often changed by some influencing conditions such as heat, light, and oxidation. Therefore, colorants have been utilized to enhance or restore the color of foods. For instance, the ancients used some natural extracts to improve the product's appearance (Schweiggert, 2018).

Colorants are classified as artificial and natural by The United States Food and Drug Administration (FAD). Due to advantages of less costly, abundantly available, providing better stability and higher tinctorial strength, the former has long been a staple of food companies compared with the latter (Rodriguez‐Amaya, 2019). However, recently there has been a growing concern about the potential negative health consequences of artificial colorants including allergic reactions, neurological effects, and risk of carcinogenicity (Albuquerque et al., 2020). Meanwhile, consumers are more and more interested in healthy, environmentally friendly, and natural food (Fernandes et al., 2019). For these reasons, the safer natural colorants derived from animals or plants are starting to enter the sights of consumers and food companies.

*Gardenia jasminoides* var. *radicans* Makikno, cultivated in the central and southern regions of China, is thought as a form of *G*. *jasminoides* Ellis. Gardenia yellow pigment (GYP) extracted from *G*. *jasminoides* var. *radicans* Makikno is a rare natural water‐soluble carotenoid, which mainly composed of crocetin and crocins (Yin & Liu, 2018). It has been applied to foods, such as candy, noodles, and beverages, as a natural food colorant due to its good water solubility, low toxicity, and allergy (Bathaie et al., 2014; Xiao et al., 2017). Besides, it is characterized by strong dyeing ability, high stability, abundant nutritional value compared with other natural colorants (Yang et al., 2009). In addition, pigments responsible for yellow, orange are especially interested in food companies because they showed more widely applications than other colors of pigments (Hatzakis et al., 2019). Therefore, the demand for GYP production and purification is increasing in the international markets.

Nowadays, selecting an effective method to generate pigments is not easy, since the changes of temperature, pH, time et al may be led to degrading some compounds related with color (Gimenez et al., 2015; Li et al., 2020; Moller et al., 2020). Therefore, it is a key step to control these variables influencing the process efficiency. Combining with the variables that offer the maximum yield of the target compounds could provide an appropriate way to solve the extraction limitations of pigments based on feasible test conditions (Pinela et al., 2019). The response surface methodology (RSM) was commonly used in parameter testing and its interactive effects (Parra‐Campos & Ordonez‐Santos, 2019; Lin et al., 2018; Wang et al., 2020). To our best knowledge, there are few studies about the optimal extraction of compounds related to GYP. Sarfarazi et al. (2019) reported the extraction process of crocin pigment of saffron through subcritical water extraction (SWE). However, as one of the precious spices all over the world, saffron is too expensive to be widely applied as a colorant. Shang et al. (2019) extracted GYP from *G*. *jasminoides* Ellis by RSM. Nevertheless, *G*. *jasminoides* Ellis, as traditional Chinese medicine, is mainly studied in the effect of its antioxidant components on human health.

It has been reported that *G*. *jasminoides* var. *radicans* Makikno contains a higher amount of GYP compared with *G*. *jasminoides* Ellis (Chen et al., 2012). This study aims to optimize the extraction process of GYP from *G*. *jasminoides* var. *radicans* Makikno through heat‐aided solvent extraction and to assess its antioxidant activity (AA). For that purpose, the RSM was used to optimize processing parameters (ethanol concentration, liquid/solid ratio, extraction time, and extraction temperature). Then, a suitable macroporous resin was selected to purify GYP. Meanwhile, the major component was isolated and identified from the purified GYP.

## 2 MATERIALS AND METHODS

### 2.1 Materials and reagents

*Gardenia jasminoides* var. *radicans* Makikno was purchased in Bozhou, Anhui medicinal materials market from Bozhou City, Anhui Province, China, and authenticated by Prof. Xueshi Liu (Professor of Plant Taxonomy, College of Life Science, Anhui Agricultural University). 1, 1‐diphenyl‐2‐picrylhydrazyl (DPPH) were purchased from Fuzhou Feijing Biotechnology Co. LTD in China. All aqueous solutions were prepared with distilled water. Other reagents were of analytic grade.

### 2.2 Extraction of GYP and solvent selection

The dried gardenia fruit was crushed by a flour‐mixing machine and the powder granularity was 0–1 mm. 0.5 g gardenia powder and different solvents (distilled water, 60% methanol–water solution, 60% ethanol–water solution and 60% isopropanol–water solution) were added into a sealed test tube for extraction in designed extraction time, solvent concentration, liquid/solid ratio, and temperature.

### 2.3 Single‐factor experiment

The effect of every single factor on the extraction yield of GYP was evaluated by the single‐factor experiment. The initial conditions were designed as followed ethanol concentration 60%, liquid/solid ratio 12:1 ml/mg, extraction time 40 min, extraction temperature 50℃. The effect of each single‐factor (ethanol concentration, liquid/solid ratio, extraction time, extraction temperature) was tested as follows: A factor was varied in defined ranges while the other factors were kept constant in each extraction experiment. Therefore, the effect of ethanol on extraction was tested at 50%, 60%, 70%, 80%, and 90% ethanol while the other factors were kept constant. Similarly, the following effects of other factors were tested: liquid/solid ratio from 10:1 to 16:1 ml/g; extraction time from 30 to 70 min; and extraction temperature from 30 to 70℃.

### 2.4 Experimental design

The optimun conditions of the extraction process were evaluated by Box–Behnken design (BBD) of RSM. According to the results from the single‐factor experiments, the levels of coded independent variables (ethanol concentration [X_1_], liquid/solid ratio [X_2_], extraction time [X_3_], extraction temperature [X_4_]) were selected to obtain optimistic extraction conditions as shown in Table S1. A total of 29 experiments with different combinations of four factors were performed based on BBD (Table S2). The absorbance and AA of GYP were selected as the responses. A second‐order model was utilized in response surface methodology (Guo et al., 2019). The equation was expressed as follows:

$$Y=\beta_{0}+\sum_{i=1}^{4}\beta_{\mathrm{ij}}X_{i}^{2}+\sum_{i=1}^{3}\sum_{j=1+1}^{4}\beta_{\mathrm{ij}}X_{i}X_{j},$$

where Y is the predicted response; β_0,_ β*_i_*, β*_ii_*, β*_ij_* $\beta_{0,}\beta_{i,}\beta_{ii,}\beta_{\mathrm{ij}}$are constant coefficients of intercept, linear, quadratic, and interactive terms, respectively. *X_i,_*, *X_j_* $X_{i}X_{j}$are levels of the coded independent variables.

### 2.5 Determination of GYP absorbance

After the extraction, the obtained solutions of GYP were centrifuged at 4,500 r/min for 5 min. 1 ml supernatant and same concentration aqueous ethanol were transferred into a 100 ml volumetric flask and then the final volume was adjusted to 100 ml. Aqueous ethanol was used as a contrast, and the absorbance of the sample solution was determined at 440 nm by UV‐Vis spectrophotometer (Zhu et al., 2014).

### 2.6 DPPH antioxidant assay

The DPPH scavenging activity for the tested sample was performed according to method detailed elsewhere with slight modifications (Sharmila et al., 2019). Briefly, 1 ml methanolic DPPH solution (0.0109 g in 100 ml methanol), 3 ml methanol, and 50 μl sample (pigment extraction) were mixed on the 10 ml tubes. 50 μl methanol was added to the test tube instead of the sample as the control. Then, the tubes were left in a dark place at room temperature for 30 min. The absorbance was determined at 517 nm using UV‐Vis spectrophotometer. The scavenging activity was evaluated by the following formula:

$$\text{Antioxidant}\;\text{activity}\;\left(\%\right)\;=\;\left(A_{\text{control}}\;‐\;A_{\text{sample}}\right)\;/\;A_{\text{control}}\;\times \;100\%,$$

where A_control_ is the absorbance of the DPPH solution and A_sample_ is the absorbance of the sample.

### 2.7 GYP purification by the macroporous resin

Macroporous resins including HPD‐400, HPD‐450, LSA‐10, AB‐8, HPD‐722, HPD‐300, HPD‐750, D‐101, LX‐11, HPD‐400A, HPD‐826, HPD‐100A were provided from Cang Zhou Bon Adsorber Technology Co., Ltd.. BJ‐7514 was bought from Jiangsu Jinkai Chemical Industry. Their physical properties are summarized in Table S3. The pretreatment of macroporous resin was described according to the previous method (Pan et al., 2017; Zhang et al., 2011) as follows: macroporous resins were first soaked in 95% ethanol for 24 hr, and then washed with distilled water until no alcohol taste. Next, the resins were pre‐treated with 5% HCl and 2% NaOH solutions to remove salts and impurities. Finally, the resins were washed to neutrality with distilled water and dried in a vacuum at 60℃.

#### 2.7.1 Adsorption of macroporous resin on pigment

The specific operations about absorption and desorption texts of GYP were described as follows: thirteen different dry resins (1.0 g) were mixed with aliquots (50 ml) of diluted pigment solution in a 250‐ml conical flask with a lid, respectively. Then, the flasks were put into a shaker and continued to shake (120 rpm) at 28℃ for 24 hr. The absorbance of the solution at 440 nm was determined by UV/Vis spectrophotometer. The adsorption rate of pigment was calculated using the following equations:

$$\text{Ar}\;\left(\%\right)\;=\;\left[\left(A_{0}‐A_{1}\right)/A_{0}\right]\;\times \;100\%,$$

where A_0_ is the absorbance of pigment solution before adsorption, A_1_ is the absorbance of pigment solution after absorption, Ar is the adsorption rate of sample (%).

#### 2.7.2 The effect of different ethanol concentrations on desorption

After the adsorption equilibrium of 1.00 g macroporous resin BJ‐7514 was reached, the residual pigment on the surface of the resin was removed by washing with distilled water. The resins and 100 ml different concentrations of ethanol–water solution (10%, 30%, 70%, 80%, 90%, 100% respectively) were added in the 250‐ml conical flask with a lid to shake (120 rpm) at 28 ℃ for 24 hr. Desorption rate was calculated by the following equation:

$$\text{Dr}\;\left(\%\right)\;=\;[\left(A_{2}/\left(A_{0}\;‐\;A_{1}\right)\right]\;\times \;100\%,$$

where A_2_ is the absorbance of the pigment–ethanol solution after desorption and Dr is the desorption rate of the test sample (%).

### 2.8 Isolation and characterization of the major compound from GYP

The refined GYP aqueous solution was partitioned with ethyl acetate, which was concentrated under reduced pressure at 55℃ to remove organic reagent. The ethyl acetate fraction was separated by ordinary‐phase silica gel eluted with CH_2_Cl_2_‐MeOH (v/v; 100:0, 100:1, 100:2, 100:4, 100:8, 100:16, and 0:100) to afford seven fractions (F1‐F7) monitored by the TLC. The Fraction 6 was precipitated in MeOH to gain crocin‐3 as red powder.

The structure of compound was identified by spectroscopic analysis. The NMR spectra were recorded at 25℃ with Agilent 600 MHz DD2 spectrometer NMR. The HR‐ESI‐MS spectra were recorded on Agilent‐1260/6460 mass spectrometer.

## 3 RESULTS AND DISCUSSION

### 3.1 Comparison with different types of solvents

The solubility of GYP in different polar solvents was significantly different. Therefore, choosing a suitable solvent could improve the extraction amount of GYP (Rammuni et al., 2019). As shown in Fig. S1, the maximum absorbance was obtained with ethanol–water solution (0.835) followed by isopropanol–water solution (0.687), methanol–water solution (0.631) and water (0.457). The result is similar with Sharmila et al. (2019) that reported the organic solvent combined with water may facilitate better extraction solvents of pigments that are soluble in water and organic solvent. So, ethanol–water solution was selected as the ideal extraction solvent for further study.

### 3.2 Single‐factor experiment of GYP extraction

Single‐factor experiments of GYP extraction were carried out with four selected parameters including ethanol concentration, liquid/solid ratio, extraction time, extraction temperature, which provided a suitable range for the BBD (Gao et al., 2017).

**FIGURE 1:**
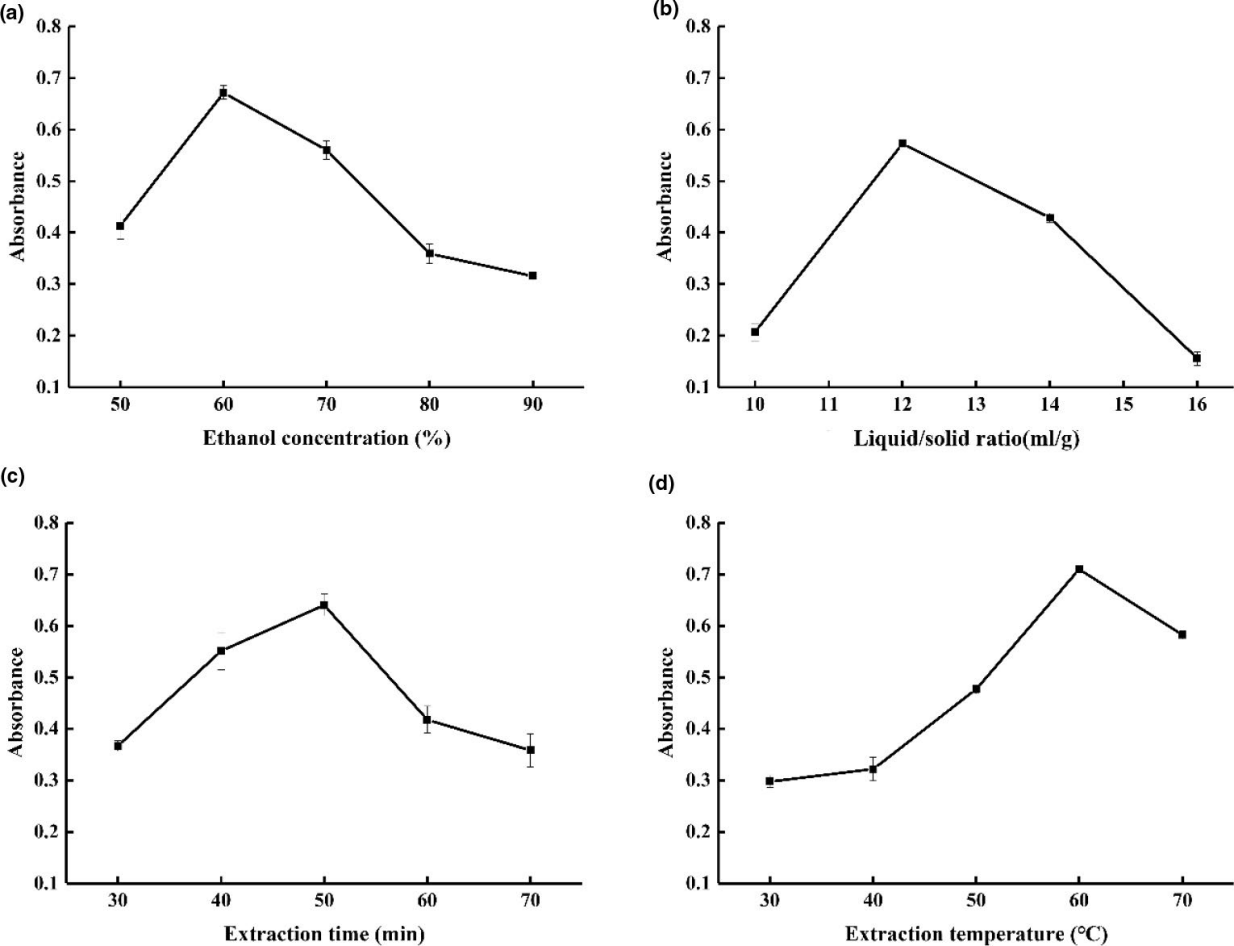
The effect of ethanol concentration (a), liquid/solid ratio (b), extraction time (c), and extraction temperature (d) on the absorbance of gardenia yellow pigment

From Figure 1a, the absorbance of GYP first increased with the ethanol concentration from 50% to 60%, then fell when the ethanol concentration was in the range of 60%–90%. The highest absorbance of GYP was 0.672 with the ethanol concentration of 60%. The result was similar to that reported by Liu, Luo, Wang, and Yuan (2019), who found low ethanol concentration enhanced the yield of the extracts. It also indicated most of the compounds connected with GYP may have high‐polarity. The similar change trend appeared for the influences of liquid/solid ratio, extraction time, and extraction temperature (Figure 1b–d). The absorbance of GYP reached the maximum when liquid/solid ratio, extraction time, and extraction temperature reached 12:1 ml/g, 50 min, and 60℃, respectively. Suitable extraction time and extraction temperature can benefit to the mass transfer to the solution. However, excessive extraction time and extraction temperature may be attributed to the degradation of pigments, such as oxidation or pyrolysis to form other compounds (Boon et al., 2010; Carmona et al., 2006).

### 3.3 Optimization of extraction by RSM

The RSM can analyze the interactive effects of all variables and provide the optimal conditions based on the tested ranges. It is a special consideration to obtain the best ranges of the relevant variables through an in‐depth extraction. Backes et al. (2018) reported that non‐best ranges of solid–liquid extraction may lead to incorrect conclusions from RSM. Thus, this experimental design of process optimization by using RSM with BBD was ground on the foregoing results of the single‐factor test.

#### 3.3.1 Analysis of the theoretical response surface model

This study was to optimize the extraction conditions of GYP from *G*. *jasminoides* var. *radicans* Makikno and to assess the antioxidant activity of the extracted pigment. Based on the experimental results of BBD and regression analysis, a quadratic polynomial equation was established to identify the relationship between the variables and responses such as absorbance (Y_1_) and AA (Y_2_). A total of 29 experiments with different combinations of four factors were performed based on BBD in Table S2. The response values ranged from 0.55 to 0.82 of absorbance and from 25.57% to 87.96% of AA. The absorbance value of 0.82 and AA of 87.96% were observed to be maximum response values from runs 29 and 13, respectively. These equations were expressed as followed:

Y_1_ = 0.80 – 0.005333X_1_ ‐ 0.041X_2_ + 0.008667X_3_ + 0.00825X_4_ + 0.00075X_1_X_2_ + 0.018 X_1_X_3_ + 0.028X_1_X_4_ ‐ 0.047X_2_X_3_ ‐ 0.016X_2_X_4_ + 0.014X_3_X_4_ ‐ 0.060$X_{1}^{2}$ ‐ 0.024$X_{2}^{2}$ ‐ 0.082$X_{3}^{2}$‐0.13$X_{4}^{2}$.

Y_2_ = 61.71 + 0.92X_1_ – 8.79X_2_ + 1.31X_3_ + 1.17X_4_ –3.83X_1_X_2_ + 0.47 X_1_X_3_ –0.82X_1_X_4_ – 0.35X_2_X_3_ + 2.89X_2_X_4_ – 11.93X_3_X_4_ + 8.06$X_{1}^{2}$ + 2.33$X_{2}^{2}$ + 12.51$X_{3}^{2}$ –17.80$X_{4}^{2}$.

X_1_, X_2_, X_4_, and X_3_ are the values of four independent variables (ethanol concentration, liquid/solid ratio, extraction time, extraction temperature).

In general, it is statistical significant because of *p*‐value with <.05. As shown in Tables 1, 2, the ANOVA presented that all the models were significant (*p*‐value < .05), and lack of fit was not significant with *p*‐value of .0544 (>.05). Therefore, the quadratic model was fitted well to the data of the experiment by ANOVA (Zhang et al., 2013). From Table 1, the linear effect of X_2_ (liquid/solid ratio)_,_ the square effect of $X_{1}^{2}$ (ethanol concentration), $X_{3}^{2}$ (extraction time), $X_{4}^{2}$ (extraction temperature) , and the interaction of X_2_X_3_ (liquid/solid ratio versus extraction time) were significant for the absorbance of GYP. Similarly, the linear effect of X_2_ (liquid/solid ratio), the square effect of $X_{3}^{2}$ (extraction time), $X_{4}^{2}$ (extraction temperature), and the interaction of X_3_X_4_ (extraction time vs. extraction temperature) had significant effect for antioxidant activity (AA, Table 2).

**Table 1:** Analysis of mean square deviation of regress equation for the absorbance of GYP

Source	Sum of squares	*df*	Mean squares	*F*‐Value	Prob > *F*
Model	0.18	14	0.013	17.86	<.0001
X_1_	3.41E‐04	1	3.41E‐04	0.49	.4964
X_2_	0.02	1	0.02	28.47	.0001
X_3_	9.01E‐04	1	9.01E‐04	1.29	.2755
X_4_	8.17E‐04	1	8.17E‐04	1.17	.2983
X_1_X_2_	2.25E‐06	1	2.25E‐06	3.21E‐03	.9556
X_1_X_3_	1.26E‐03	1	1.26E‐03	1.8	.201
X_1_X_4_	3.03E‐03	1	3.03E‐03	4.32	.0565
X_2_X_3_	9.03E‐03	1	9.03E‐03	12.89	.003
X_2_X_4_	1.09E‐03	1	1.09E‐03	1.56	.2328
X_3_X_4_	8.12E‐04	1	8.12E‐04	1.16	.2996
$X_{1}^{2}$	0.023	1	0.023	33.02	<.0001
$X_{2}^{2}$	3.60E‐03	1	3.60E‐03	5.15	.0396
$X_{3}^{2}$	0.044	1	0.044	62.98	<.0001
$X_{4}^{2}$	0.11	1	0.11	156.16	<.0001
Residual	9.80E‐03	14	7.00E‐04	–	–
Lack of fit	9.16E‐03	10	9.16E‐04	5.68	.0544
Pure error	6.45E‐04	4	1.61E‐04	–	–
Cor total	0.18	28	–	–	–
*R* ^2^ = .9470					

*df*, degrees of freedom; *F*‐value, Fischer test value; *p*, probability value; *R* ^2^, determination coefficient.

**Table 2:** Analysis of mean square deviation of regress equation for antioxidant activity (AA) of GYP

Source	Sum of squares	*df*	Mean squares	*F*‐value	Prob > *F*
Model	5,965.73	14	426.12	4.17	.0058
X_1_	10.21	1	10.21	0.100	.7565
X_2_	926.82	1	926.82	9.07	.0093
X_3_	20.51	1	20.51	0.20	.6609
X_4_	16.43	1	16.43	0.16	.6944
X_1_X_2_	58.68	1	58.68	0.57	.4610
X_1_X_3_	0.86	1	0.86	8.468E‐003	.9280
X_1_X_4_	2.71	1	2.71	0.026	.8730
X_2_X_3_	0.49	1	0.49	4.798E‐003	.9458
X_2_X_4_	33.29	1	33.29	0.33	.5771
X_3_X_4_	569.06	1	569.06	5.57	.0333
$X_{1}^{2}$	421.85	1	421.85	4.13	.0615
$X_{2}^{2}$	35.23	1	35.23	0.34	.5663
$X_{3}^{2}$	1,015.85	1	1,015.85	9.95	.0070
$X_{4}^{2}$	2055.60	1	2055.60	20.13	.0005
Residual	1,429.86	14	102.13		
Lack of fit	1,181.47	10	118.15	1.90	.2803
Pure error	248.39	4	62.10		
Cor total	7,395.60	28			
*R* ^2^ = .8067					

*df*, degrees of freedom; *F*‐value, Fischer test value; *p*, probability value; *R* ^2^‐determination coefficient.

#### 3.3.2 The interaction between the independent variables

**FIGURE 2:**
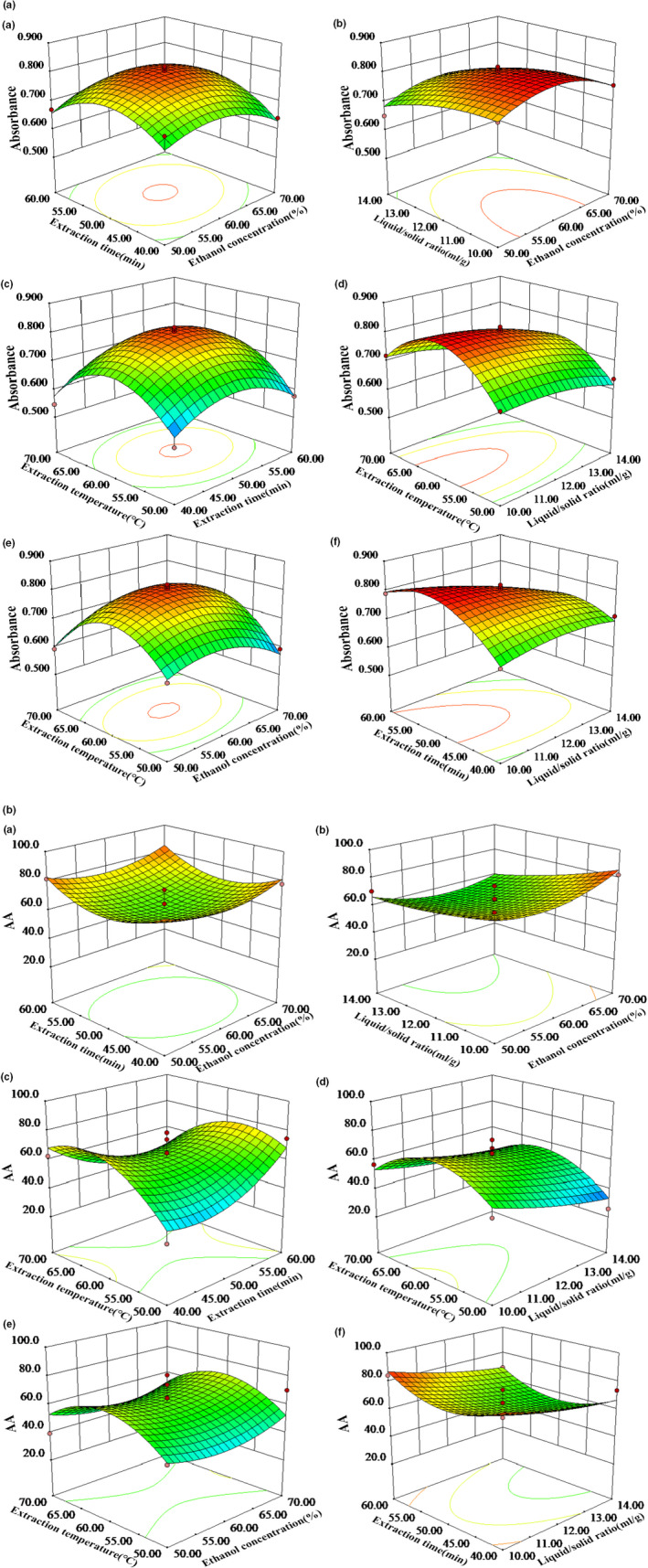
The three‐dimensional plot showing the correlative effects of extraction time and ethanol concentration (a), extraction liquid/solid ratio and ethanol concentration (b), extraction temperature and time (c), extraction temperature and liquid/solid ratio (d), extraction temperature and ethanol concentration (e) and extraction time and liquid/solid ratio (f) on the value of absorbance of GYP (part A) and the AA of GYP (part B)

The three‐dimensional (3D) response surface curves denoted the interaction between the independent variables and ensured the optimal levels of each variable for the maximum absorbance and AA (da Silva et al., 2019). As shown in Figure 2, two variables remained unchanged, while the other two variables changed within the defined range. The three‐dimensional (3D) response surface curves (Figure 2, Part A, a‐f) illustrated the effects of the interaction between the independent variables in the absorbance of GYP. The significant negative linear effect of X_2_ (liquid/solid ratio) and the negative square effect of $X_{3}^{2}$ (ethanol concentration), $X_{3}^{2}$ (extraction time), $X_{4}^{2}$ (extraction temperature) in the absorbance were presented in Figure 2, Part A. Therefore, the maximal absorbance was obtained at the low level of liquid/solid ratio (10 ml/g), the middle level of ethanol concentration (60%), the middle level of extraction time (50 min), the middle level of extraction temperature (60 ℃).

The effects of the four independent variables on the response value (AA) were visually described by the 3‐D plot (Figure 2, Part B, a–f). The results showed that the low level of liquid/solid ratio (10 ml/g), the middle level of ethanol concentration (60%), the middle level of extraction time (50 min), the middle level of extraction temperature (60℃), led to the optimal results.

#### 3.3.3 Verification of predictive model

Optimal conditions were predicted to get to the maximal value of absorbance (0.79) and AA (91.2%) by RSM with ethanol concentration 65.1%, liquid/solid ratio 10:1 ml/g, extraction time 59.8 min and extraction temperature 60.0℃.

To verify the optimal values of extraction conditions predicted by RSM, the real experiment was operated under the optimal conditions. Ultimately, the real experimental values were absorbance (0.78) and AA (84.6%), which were well matched with values predicted by the regression model. Therefore, the extraction conditions acquired by RSM were practical.

### 3.4 GYP purification by the macroporous resin

The crude GYP may contain other compounds that not related to the yellow pigment, such as iridoids, quinic acid derivatives, flavonoids, triterpenoids, and organic esters (Wang et al., 2016). Some of these compounds, especially the colorless geniposide and chlorogenic acid, could cause crude GYP to get green or darkened. Thus, it is a necessary step to select an effective method to purify crude GYP. The absorption method by macroporous resins is frequently considered to be low cost, easy to operate, and high purity (Yang et al., 2009).

#### 3.4.1 Absorption effect of macroporous resin on the pigment

**FIGURE 3:**
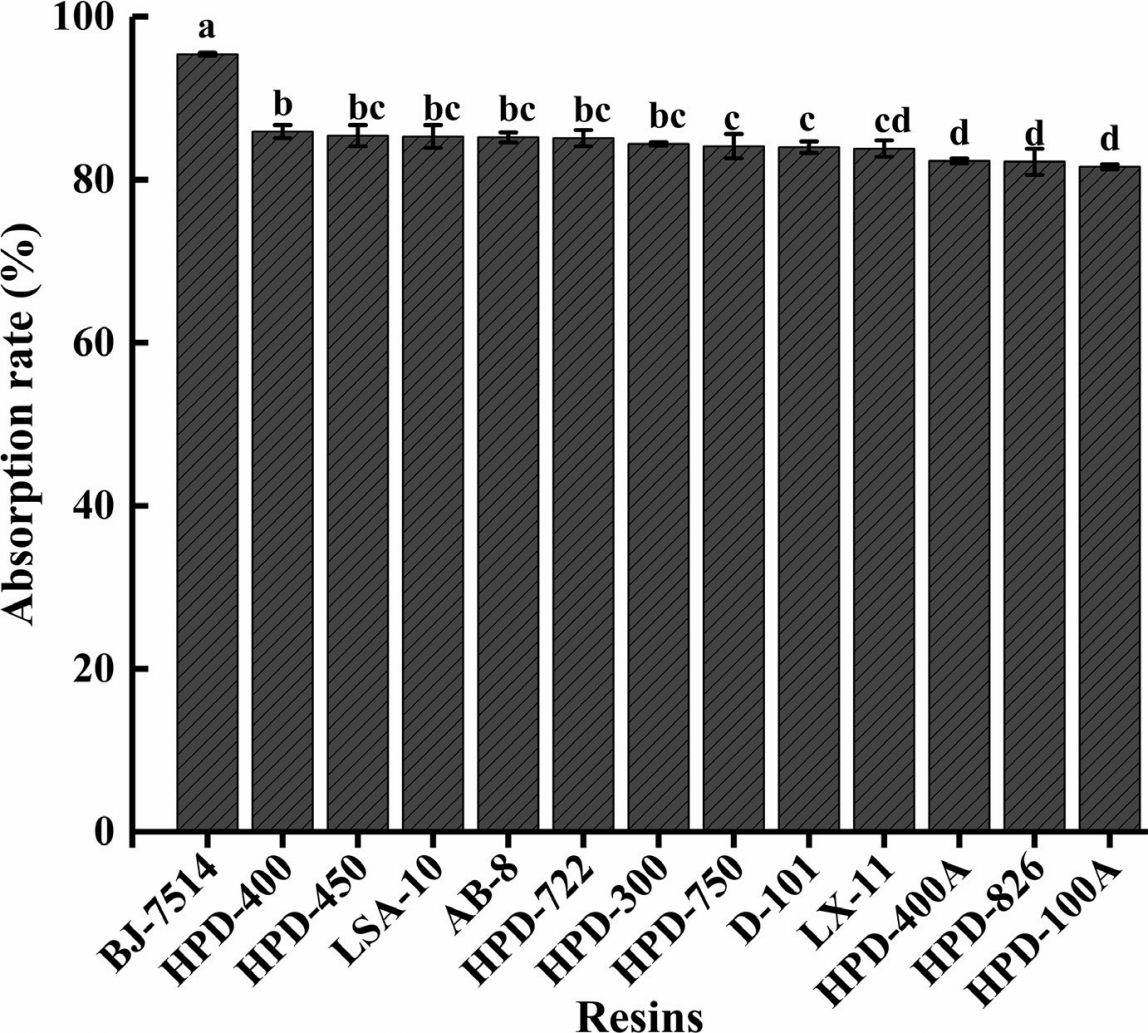
Adsorption ratios of yellow pigment on different resins

Thirteen different properties of macroporous resins were used to screen the most effective one for the purification of GYP. As showed in Figure 3, the highest absorption ratios of GYP was 95.4% on macroporous resin of BJ‐7514. It may be attributed to acrylic acid of BJ‐7514 possesses the stronger capacity to bond with GYP contained crocetin and crocins compared with other resins regarded polystyrene as a functional group. Therefore, BJ‐7514 was thought of as a potential resin to purify GYP from *G*.* jasminoides* var. *radicans* Makikno.

#### 3.4.2 Effect of different concentration of ethanol solution on desorption

Macroporous resins are made of polymers that possess pores and larger surface areas. The principle of the target compound's surface adsorption could be achieved by the formation of physical and/or chemical bonds. And the compounds adsorbed onto the resin surface must be desorbed by using an eluting solvent that can destroy the bonds between them (Belwal et al., 2020). Different concentrations of ethanol solution were tested to find a suitable elution to purify the crude pigment. The desorption rate with different concentrations of ethanol solution (10%, 30%, 70%, 80%, 90%, 100%, respectively) on the resins was shown in Fig. S2. It was clear to observe that the ethanol solution (80%) presented the highest desorption rate (91.93%) on the crude pigment.

### 3.5 Structure elucidation of the major compound from GYP

The major compound of the purified GYP was identified as crocin‐3 (2.74% yield rate) (Fig S3) by spectroscopic analyses, including HR‐ESI‐MS and NMR, and comparisons with published literature (Van et al., 1997).

Crocin‐3: Red powder (MeOH); HR‐ESI‐MS: m/z 675.2629 [M + Na]^+^, indicated for C_32_H_44_O_14_Na; ^1^H NMR (600 MHz, DMSO‐d6): δ 5.42 (1H, d, J = 7.7, H‐1), δ 2.95 – 3.45 (1H, m, H‐2 ‐ H‐5), δ 3.56 – 3.65 (1H, m, H‐6), δ 7.35 (1H, d, J = 12.3, H‐10), δ 6.65 (1H, m, H‐11), δ 6.85 (1H, m, H‐12), δ 6.49 (1H, m, H‐14), δ 6.87 (1H, m, H‐15), δ 1.97 (1H, s, H‐19), δ 1.98 (1H, s, H‐20), δ 4.16 (1H, d, J = 7.8, H‐1′), δ 2.95 – 3.45 (1H, m, H‐2′ ‐ H‐5′), δ 3.56 – 3.65 (1H, m, H‐6′), δ 7.20 (1H, d, J = 11.2, H‐10′), δ 6.72 (1H, m, H‐11′), δ 6.53 (1H, m, H‐12′), δ 6.51 (1H, m, H‐14′), δ 6.76 (1H, m, H‐15′), δ 1.92 (1H, s, H‐19′), δ 1.92 (1H, s, H‐20′); ^13^C NMR (150 MHz, DMSO‐d6): 95.0 (C‐1), 72.9 (C‐2), 76.7 (C‐3), 69.8 (C‐4), 77.3 (C‐5), 68.4 (C‐6), 166.4 (C‐8), 124.6 (C‐9), 140.3 (C‐10), 125.7 (C‐11), 145.0 (C‐12), 137.3 (C‐13), 136.4 (C‐14), 132.4 (C‐15), 13.0 (C‐19), 13.0 (C‐20), 103.6 (C‐1′), 73.9 (C‐2′), 76.8 (C‐3′), 70.5 (C‐4′), 77.2 (C‐5′), 61.5 (C‐6′), 166.4 (C‐8′), 124.2 (C‐9′), 138.4 (C‐10′), 131.96 (C‐11′), 143.6 (C‐12′), 138.2 (C‐13′), 135.6 (C‐14′), 132.0 (C‐15′), 12.9 (C‐19′), 13.0 (C‐20′).

Crocin‐3 belongs to crocetin derivatives contained one gentiobiosyl. It was widely concerned due to its healthy benefits such as neuro‐protection, anti‐depression, and antioxidant activity (Dar et al., 2017; Lv et al., 2016).

## 4 CONCLUSION

The current work described the optimum extraction process and purification of GYP from *G*. *jasminoides* var. *radicans* Makikno. The optimum extraction conditions were determined by RSM based on single‐factor experiments. The results showed that the optimal extraction conditions were ethanol concentration (65.10%), liquid/solid ratio (10:1 ml/g), extraction time (59.85 min), and extraction temperature (60.04℃) for the maximal response values of absorbance (0.79) and AA (91.30%), respectively. BJ‐7514 was screened out as the most suitable resin to purify the crude GYP. Moreover, the 80% of ethanol eluent was applicable on the BJ‐7514 to obtain the purified GYP. In addition, the major component of GYP (Crocin‐3) was isolated and identified from the purified GYP. This study will be a prospect for the application of GYP from *G*.* jasminoides* var. *radicans* Makikno.

## 6 CONFLICT OF INTEREST

None.

7

## Supporting information

Supplementary Material

## Data Availability

The data that supports the findings of this study are available in the supplementary material of this article.
