# Supplementary material for: Extraction optimization by using response surface methodology and purification of yellow pigment from Gardenia jasminoides var. radicans Makikno
Source: Food Sci Nutr. 2020 Dec 4;9(2):822–32. doi: 10.1002/fsn3.2046 (PMC7866593; doi:10.1002/fsn3.2046)
Supplement: Supplementary file 1 — Supplementary Material [file FSN3-9-822-s001.docx]

Supplementary Material

**Table S1.** Analytical factor and levels for RSM.

**Table S2.** Box-Behnken design for extraction of yellow pigment by heat aided with the observed and predicted responses.

**Table S3.** Physical characteristics of the tested macroporous resins.

**Fig. S1.** Effect of concentration is 60% of the different solvent types on the absorbance of gardenia yellow pigment.

**Fig. S2.** Effect of ethanol concentration on desorption of macroporous resin.

**Fig. S3.** Chemical structure of crocin-3 from *Gardenia jasminoides* var. *radicans* Makikno.

**Table S1.** Analytical factor and levels for RSM.

| Independent variables | Levels | | |
| --- | --- | --- | --- |
|  | -1 | 0 | 1 |
| Ethanol concentration (%, X_1_) | 50 | 60 | 70 |
| Liquid/solid ratio (ml/g, X_2_) | 10:1 | 12:1 | 14:1 |
| Extraction time (min, X_3_) | 40 | 50 | 60 |
| Extraction temperature (℃, X_4_) | 50 | 60 | 70 |

**Table S2.** Box-Behnken design for extraction of yellow pigment by heat aided with the observed and predicted responses.

| std | X1:  Ethanol concentration (%) | X2:  Liquid/solid ratio (ml/g) | X3:  Extraction time (min) | X4:  Extraction temperature (℃) | Response1 Absorbance | | Response2  AA (%) | |
| --- | --- | --- | --- | --- | --- | --- | --- | --- |
|  |  |  |  |  | Observed | Predicted | Observed | Predicted |
| 1 | 50 | 10 | 50 | 60 | 0.77 | 0.77 | 81.26 | 76.14 |
| 2 | 70 | 10 | 50 | 60 | 0.76 | 0.76 | 82.08 | 85.65 |
| 3 | 50 | 14 | 50 | 60 | 0.65 | 0.68 | 70.18 | 66.23 |
| 4 | 70 | 14 | 50 | 60 | 0.64 | 0.68 | 55.68 | 60.41 |
| 5 | 60 | 12 | 40 | 50 | 0.56 | 0.59 | 33.66 | 42.02 |
| 6 | 60 | 12 | 60 | 50 | 0.58 | 0.58 | 74.57 | 68.49 |
| 7 | 60 | 12 | 40 | 70 | 0.55 | 0.58 | 62.52 | 68.21 |
| 8 | 60 | 12 | 60 | 70 | 0.62 | 0.62 | 55.72 | 46.97 |
| 9 | 50 | 12 | 50 | 50 | 0.63 | 0.64 | 48.01 | 49.06 |
| 10 | 70 | 12 | 50 | 50 | 0.59 | 0.57 | 70.17 | 52.55 |
| 11 | 50 | 12 | 50 | 70 | 0.59 | 0.60 | 39.31 | 53.04 |
| 12 | 70 | 12 | 50 | 70 | 0.67 | 0.64 | 58.18 | 53.24 |
| 13 | 60 | 10 | 40 | 60 | 0.68 | 0.68 | 87.96 | 83.69 |
| 14 | 60 | 14 | 40 | 60 | 0.71 | 0.70 | 73.61 | 66.81 |
| 15 | 60 | 10 | 60 | 60 | 0.79 | 0.79 | 84.09 | 87.00 |
| 16 | 60 | 14 | 60 | 60 | 0.64 | 0.62 | 68.34 | 68.73 |
| 17 | 50 | 12 | 40 | 60 | 0.72 | 0.68 | 87.01 | 80.53 |
| 18 | 70 | 12 | 40 | 60 | 0.64 | 0.63 | 77.94 | 81.44 |
| 19 | 50 | 12 | 60 | 60 | 0.67 | 0.66 | 81.44 | 82.21 |
| 20 | 70 | 12 | 60 | 60 | 0.66 | 0.68 | 74.23 | 84.99 |
| 21 | 60 | 10 | 50 | 50 | 0.68 | 0.67 | 50.28 | 56.74 |
| 22 | 60 | 14 | 50 | 50 | 0.64 | 0.62 | 25.57 | 33.40 |
| 23 | 60 | 10 | 50 | 70 | 0.72 | 0.72 | 56.87 | 53.31 |
| 24 | 60 | 14 | 50 | 70 | 0.61 | 0.60 | 43.70 | 41.51 |
| 25 | 60 | 12 | 50 | 60 | 0.79 | 0.80 | 57.57 | 61.71 |
| 26 | 60 | 12 | 50 | 60 | 0.79 | 0.80 | 58.28 | 61.71 |
| 27 | 60 | 12 | 50 | 60 | 0.81 | 0.80 | 74.00 | 61.71 |
| 28 | 60 | 12 | 50 | 60 | 0.81 | 0.80 | 54.00 | 61.71 |
| 29 | 60 | 12 | 50 | 60 | 0.82 | 0.80 | 64.71 | 61.71 |

**Table S3.** Physical characteristics of the tested macroporous resins.

| Name | Functional group | Polarity | Particle diameter (mm) | Average pore  diameter (Å) | | Surface area (m^2^/g) |
| --- | --- | --- | --- | --- | --- | --- |
| HPD-400 | Polystyrene | Middle-polar | 0.3–1.2 | 75–80 |  | 500-550 |
| HPD-450 | Polystyrene | Middle-polar | 0.3-1.25 | 90-110 |  | 500-550 |
| LSA-10 | Polystyrene | Middle-polar | 0.3-1.2 | 84-94 |  | 500-540 |
| AB-8 | Polystyrene | Weak-polar | 0.3-1.25 | 130-140 |  | 480-520 |
| HPD-722 | Polystyrene | Weak-polar | 0.3-1.25 | 130-140 |  | 485-530 |
| HPD-300 | Polystyrene | Non-polar | 0.3-1.2 | 50-55 |  | 800-870 |
| HPD-750 | Polystyrene | Middle-polar | 0.3-1.2 | 85-90 |  | 650-700 |
| D-101 | Polystyrene | Non-polar | 0.3-1.25 | 90-100 |  | 500-550 |
| LX-11 | Polystyrene | Non-polar | 0.3-1.25 | 25-28 |  | 480-520 |
| HPD-400A | Polystyrene | Middle-polar | 0.25-0.85 | 85-90 |  | 500-550 |
| HPD-826 | Polystyrene | hydrogen bond | 0.3-1.25 | 90-100 |  | 500-600 |
| HPD-100A | Polystyrene | Non-polar | 0.25-0.85 | 95-100 |  | 650-700 |
| BJ-7514 | [Acrylic](javascript:;) | Middle-polar | 0.6 | 150 |  | 450 |


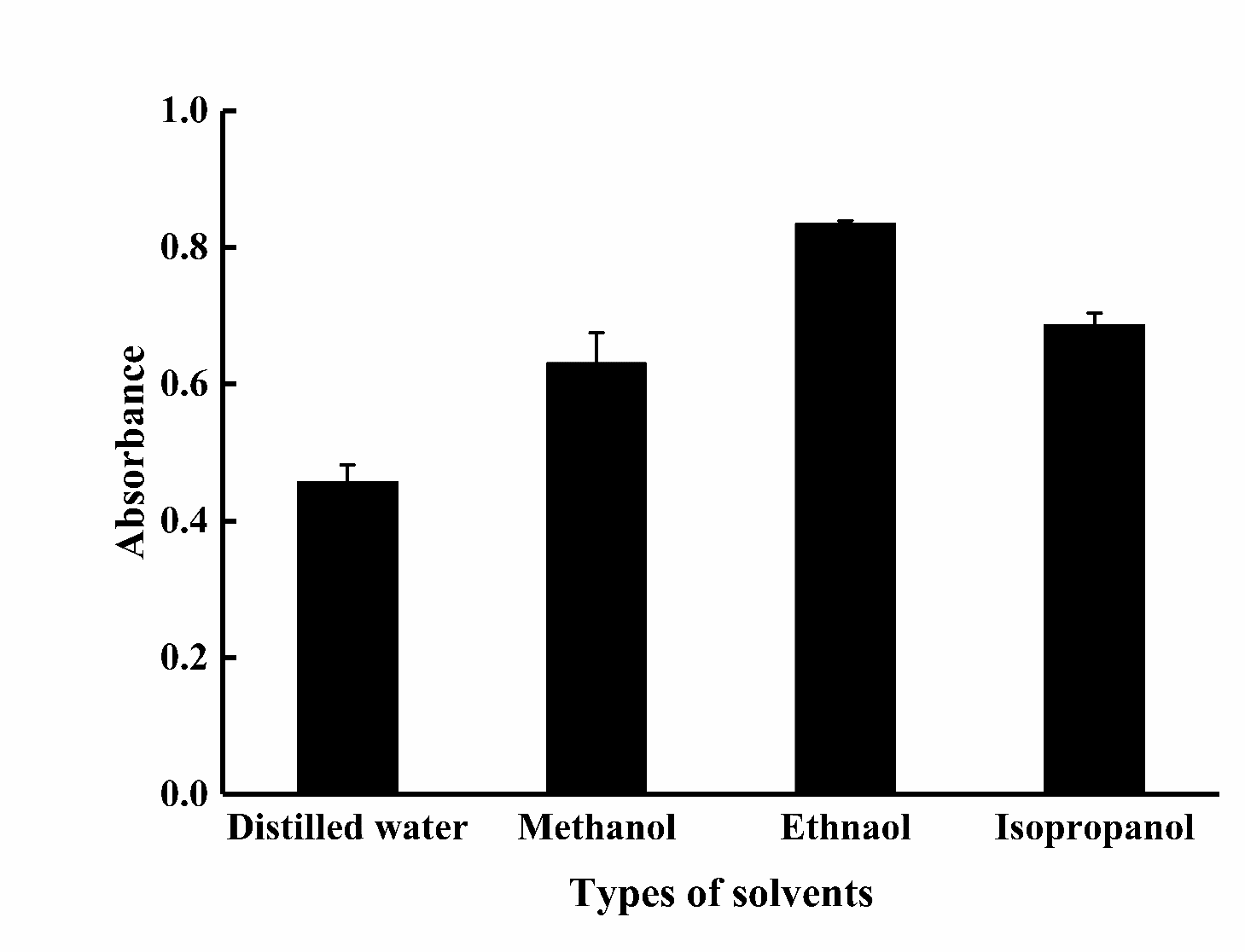


**Fig. S1.** Effect of concentration is 60% of the different solvent types on the absorbance of gardenia yellow pigment (n=3).





**Fig. S2.** Effect of ethanol concentration on desorption of macroporous resin. Results are means ± SD of three parallel measurements.


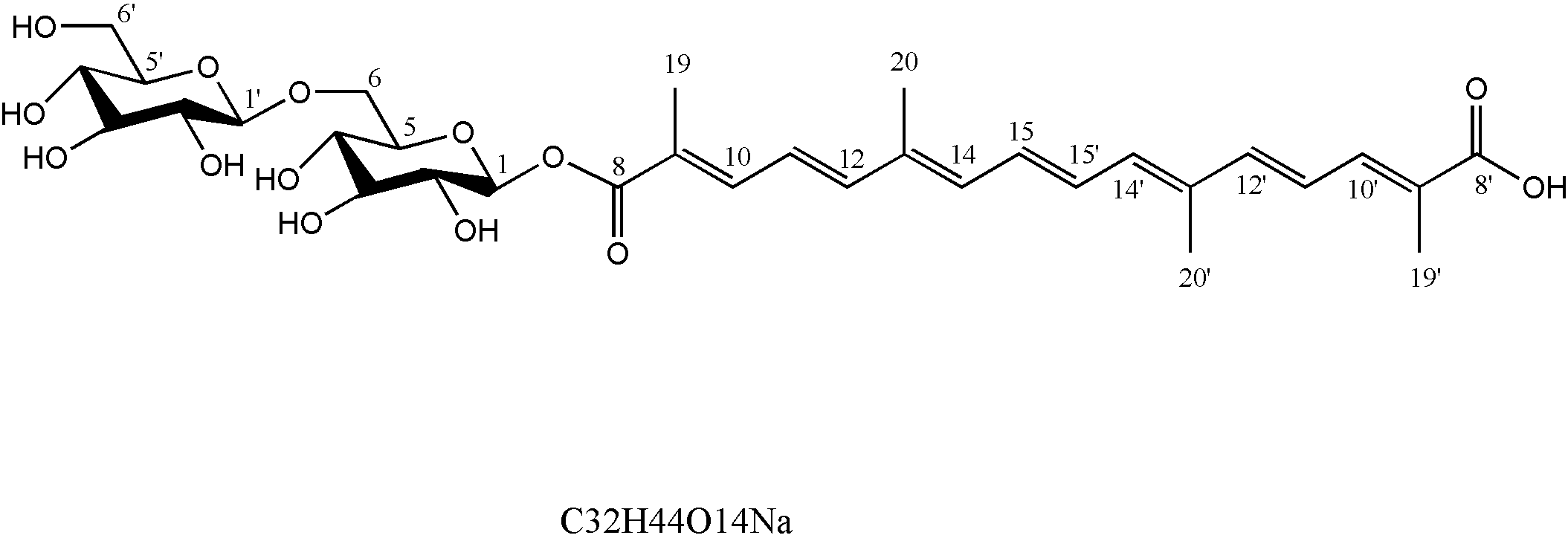


**Fig. S3.** Chemical structure of crocin-3 from *Gardenia jasminoides* var. *radicans* Makikno.
